# Sensitive Metal-Semiconductor Nanothermocouple Fabricated by FIB to Investigate Laser Beams with Nanometer Spatial Resolution

**DOI:** 10.3390/s22010287

**Published:** 2021-12-31

**Authors:** Adam Łaszcz, Andrzej Czerwinski, Emilia Pruszyńska-Karbownik, Marek Wzorek, Dariusz Szmigiel

**Affiliations:** 1 Łukasiewicz Research Network, Institute of Microelectronics and Photonics, Al. Lotników 32/46, 02-668 Warsaw, Poland; andrzej.czerwinski@imif.lukasiewicz.gov.pl (A.C.); marek.wzorek@imif.lukasiewicz.gov.pl (M.W.); dariusz.szmigiel@imif.lukasiewicz.gov.pl (D.S.); 2 Institute of Physics, Lodz University of Technology, Wólczańska 219, 90-924 Lodz, Poland; emilia.pruszynska-karbownik@p.lodz.pl

**Keywords:** focused ion beam (FIB), thermoelectric nanostructure, metal-semiconductor thermocouple, Seebeck coefficient, laser annealing

## Abstract

The focused ion beam (FIB) technique was used to fabricate a nanothermocouple (with a 90 nm wide nanojunction) based on a metal–semiconductor (Pt–Si) structure, which showed a sensitivity up to 10 times larger (with Seebeck coefficient up to 140 µV/K) than typical metal–metal nanothermocouples. In contrast to the fabrication of nanothermocouples which requires a high-tech semiconductor manufacturing line with sophisticated fabrication techniques, environment, and advanced equipment, FIB systems are available in many research laboratories without the need for a high-tech environment, and the described processing is performed relatively quickly by a single operator. The linear response of the manufactured nanothermocouple enabled sensitive measurements even with small changes of temperature when heated with a stream of hot air. A nonlinear response of the nanothermocouple (up to 83.85 mV) was observed during the exposition to an argon-laser beam with a high optical power density (up to 17.4 Wcm^−2^), which was also used for the laser annealing of metal–semiconductor interfaces. The analysis of the results implies the application of such nanothermocouples, especially for the characterization of laser beams with nanometer spatial resolution. Improvements of the FIB processing should lead to an even higher Seebeck coefficient of the nanothermocouples; e.g., in case of the availability of other suitable metal sources (e.g., Cr).

## 1. Introduction

Thermocouples are widely used as components of infrared sensors, thermal probes, motion sensors, energy generators, complex systems based on MEMS/NEMS structures, and many others [1,2,3,4,5,6]. The main advantage of thermoelectric measurement systems based on nanostructures is their very high spatial and time resolution of measurements in comparison to conventional macro or microsystems, because larger structures average the results both in space and time. This is particularly important in scientific applications and especially useful for the characterization of laser radiation, where usually microscale devices have been used so far [1,7]. For the thermoelectric energy conversion of thermal energy (heat flux) transforms to electricity, the maximum efficiency is determined by the dimensionless figure of merit (ZT) for the chosen thermoelectric materials, which is given by ZT = S2σT/κ, where S is the Seebeck coefficient, σ is the electrical conductivity, κ is the thermal conductivity, and T is the temperature. Therefore, the maximum efficiency ZT is increased by the high Seebeck coefficient as well as the low thermal conductivity and electrical resistivity of materials used for thermocouples [8].

Recent studies have shown that the nanostructuring of thermocouples can be an effective method of increasing ZT [9,10,11]. It is believed that this can be related to the reduction of the lattice thermal conductivity due to increased phonon scattering at interfaces of thermoelectric nanomaterials. From a practical point of view, the commercial thermoelectric materials commonly applied in contemporary devices usually have a relatively low Seebeck coefficient because, typically, they are metals or their alloys [2,12]. Therefore, in this study, we investigate the possibility of application of thermoelectric materials with high values of the Seebeck coefficient (e.g., semiconductors), shaped in the form of nanostructures.

In contrast to the bulk Si, the silicon nanowires or other silicon-based nanostructures have gained much attention for use in sensitive nanoscale thermoelectric systems due to their low thermal conductivity, large Seebeck coefficient, and the excellent spatial resolution of measurements. For example, n and p-type silicon nanowires demonstrated Seebeck coefficients equal to 127.6 and 141.8 μV/K, respectively, at room temperature [13] and to 170.0 and 152.8 µV/K in the temperature range from 200 to 300 K [14]. However, the application of silicon wires has not always resulted in a device with spatial nanoresolution; for example, for Si nanowire arrays with a reported high Seebeck coefficients [15,16], where the measurement results were obtained not from a single nanowire but from the whole matrix of many wires—i.e., not in the nanoscale. In addition, for a thermocouple device for bolometric applications, with poly and single-crystalline silicon wires, the reported Seebeck coefficient was high; however, none of its sizes (100 μm long and 1 μm wide) was in the nanoscale [17]. For nanothermocouples based on Cr thin film deposited on silicon, the Seebeck coefficients were equal to 924 µV/K and 515 µV/K, respectively, for nanothermocouples fabricated on the Si wafer and on the flexible Si substrate [18]. The fabrication process in the case of the above nanothermocouples [13,14,18] requires a high-tech semiconductor manufacturing line with sophisticated fabrication techniques and environments, and many pieces of specialized and advanced equipment, based on, e.g., cleanrooms, high-resolution photolithography, e-beam lithography for nanopatterns, etc. In contrast, our work shows the results of the fabrication of nanothermocouples, which lasts for a few hours, based on processing with FIB, which is available in many research laboratories (i.e., without cleanrooms and other high-tech environments) and can be performed by a single operator.

Typically, the nanothermocouples are manufactured in a multi-step process using a large amount of advanced equipment, which for obvious reasons makes the manufacturing process expensive and unavailable for many potential applications. However, the use of the FIB method in the fabrication of nanothermocouples brings new possibilities. The FIB technique is primarily dedicated to carrying out various types of technological processes in micro and nanoscales (i.e., the etching of various materials or deposition of metals and insulators), enabling the fabrication of unique structures or a modification of existing structures [19,20,21,22,23]. The simultaneous observation due to imaging with electrons and ions during FIB machining allows for the direct and precise quality control of performed FIB processes. In one experiment (without breaking vacuum), the technique allows the production of various nano or microstructures, which may be an advantage in relation to other known technologies. Moreover, the FIB systems are dedicated only to the production of small series of structures or devices—e.g., prototypes of highly specialized applications—and such instruments are particularly useful for research purposes.

The Seebeck nanojunction made of a Pt–W nanostrip prepared with the FIB technique has already been used to monitor the local temperature rise in the processed material during ion beam irradiation in FIB [24]. Although it was based on a metal–metal nanostructure, and therefore it exhibited low sensitivity to a temperature gradient, it reportedly generated a linear response of up to 3.5 mV thermovoltage with a temperature increase of about 250 °C (i.e., with a Seebeck coefficient equal to about 14 µV/K) [24].

We decided to develop the nanothermocouple with unique features and performance; for this, we used FIB fabrication and the metal–semiconductor structure. In the approach proposed in our paper, the very high sensitivity of the produced metal–semiconductor structure (not available for typical metal-metal structures) enables us to apply the nanostructure to detect even small gradients of temperature simultaneously using sensors with much reduced sizes. This results in a significant improvement in both the spatial resolution of the nanostructure in comparison to microstructures and in the sensitivity of the metal–semiconductor nanothermocouple in comparison to metal–metal thermoelectric sensors.

It is worth mentioning that also optical methods when performed for nanosized material objects result in a remarkable spatial resolution and sensitivity on detecting local temperature changes; e.g., for single silicon nanoparticles [25] or single defects in diamond [26]. Furthermore, the research into the laser beams can be performed using a near-field scanning optical microscope (NSOM) [27,28].

## 2. Materials and Methods

### 2.1. Fabrication of the Base Structure

**Figure 1:**
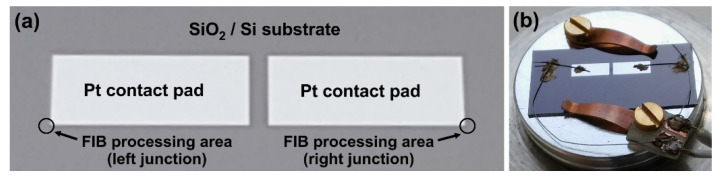
(**a**) The top-view of the base structure (with sizes of Pt contact pads equal to 2 mm × 5 mm), where the areas of thermoelectric junctions processed with FIB (marked as the left and right junction) are shown; (**b**) the base structure—already with thermoelectric junctions—prepared for the thermoelectric measurements.

The manufacturing of the nanosensor consists of the base-structure fabrication and its modification in the FIB system (Helios NanoLab 600 DualBeam) using a gallium-ion beam which forms the nanothermocouple. The base structure was manufactured on the n-type (R_SH_ ≈ 2 Ω per square) silicon substrate covered by a 500 nm thick SiO_2_ layer. The rectangular 100 nm thick platinum contact pads located in close proximity to each other (with 2 mm × 5 mm size each) were produced on the surface of this oxide using the photolithography technique (Figure 1a). Separate microwires were attached to contact pads (one for each contact pad) as electrical connections using a conductive silver paste, and those microwires were connected to separate standard electrical cables (Figure 1b).

### 2.2. Fabrication of Thermoelectric Nanostructures in the FIB/SEM System

Two thermoelectric junctions (one of the nanosize width) were manufactured in the FIB chamber using the FIB processing. These places are marked in Figure 1 as FIB processing areas of the left and right junctions. One of them is a “cold” junction (i.e., the unheated one, still at ambient temperature, also called a reference junction), and the second one is a “hot” junction (i.e., the heated one) during the measurements. They were located close to the outer edges of the contact pads, thus enabling maximum distance between the hot and cold junctions.

**Figure 2:**
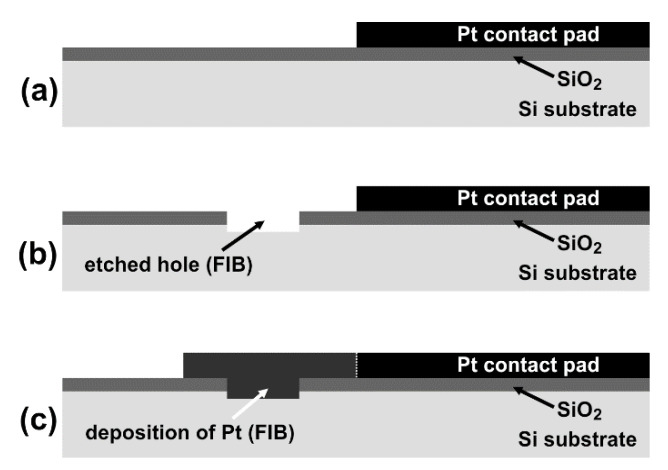
(**a**–**c**) The cross-section schemes showing consecutive steps of the FIB processing leading to the formation of a junction (e.g., the left junction) between platinum and silicon substrates. Figures (**b**,**c**) correspond to Figure 3a,b, respectively. The drawings are schematic, and the sizes are not to scale.

**Figure 3:**
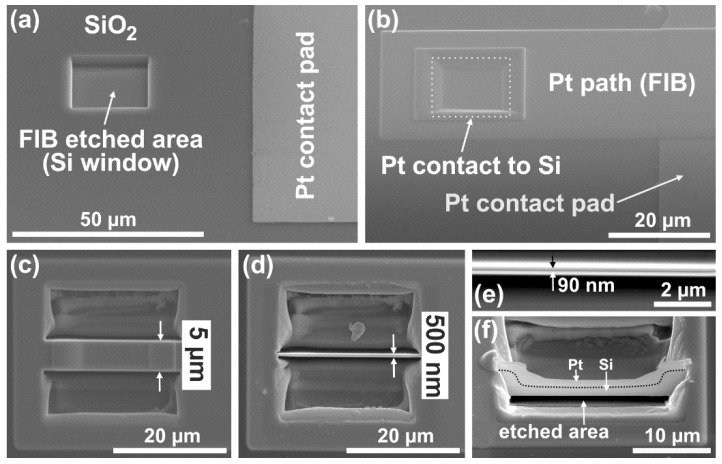
SEM images of the left structure after successive FIB operations that resulted in obtaining the thermoelectric micro or nanojunction: (**a**) after milling the window to the Si substrate through the oxide, (**b**) after deposition of Pt path aimed at covering the exposed Si (in the window) and connecting it with the Pt contact pad, (**c**) top view after milling of the deposited Pt in the window region—from its two opposite sides—to obtain a 5 µm wide thermoelectric Pt/Si junction in the middle of the hole, (**d**) top view after similar milling of the junction width to 500 nm, (**e**) the top view of the junction narrowed to 90 nm in width, and (**f**) the tilted view after removal a layer of silicon beneath this 90 nm wide junction using FIB etching process to form a bridge structure.

The idea of thermocouple fabrication is based on the concept in which an FIB deposited platinum stripe (with its width narrowed by the FIB processes) is used as the metallic material for the thermocouple junction while monocrystalline silicon substrate is used as the semiconductor material. The views after consecutive FIB operations are schematically shown in Figure 2 and presented as top-view SEM images in Figure 3. In the first step, a square 20 µm × 20 µm microhole (marked as Si-window in Figure 3a) was milled (etched) through the SiO_2_ layer to the silicon substrate using FIB. The hole was located at a distance of about 30 µm from one contact pad. In this way, access to the silicon (with one layer of the thermoelement) was provided. The next step was the FIB deposition of the platinum micropath (145 µm long, 32 µm wide, and about 1 µm thick) linking the silicon in the square microhole with the contact pad (Figure 3b). The width of the deposited Pt stripe was larger than the size of the square microhole, covering the whole area of the exposed silicon in the hole. Afterwards, the Pt layer in the region of this hole was additionally thickened using platinum deposition by FIB (about 1 µm thick Pt with a size equal to 21 µm × 21 µm, Figure 3b). In this way, one thermoelectric Pt/Si junction was fabricated. Simultaneously with these operations at one thermojunction, the other Pt/Si junction was also manufactured at the outer edge of the other contact pad using the same FIB operations and procedures described above (starting from milling the hole, as in Figure 2b and Figure 3a). The next steps led to the fabrication of the thermoelectric micro or nanostructure using gallium-ion beam milling in the area of the square hole of the left junction (Figure 3c–f). At the beginning, the process led to the fabrication of a 5 µm wide strip in the middle part of the hole area (Figure 3c). In subsequent processing steps, the width of the thermoelectric Pt/Si junction was consecutively reduced in a similar way to 2 µm, 1 µm, 500 nm (Figure 3d), 200 nm, and 90 nm (Figure 3e), respectively. The right junction was the reference junction, and its initial size was not reduced.

Measurements of thermoelectric voltage (ThV) described below were performed after each iteration after decreasing the widths of the left junction. All FIB processes leading to the fabrication of a Pt/Si micro or nanostructure were performed using an ion-beam energy of 30 kV and an ion-beam current ranging from nanoamperes to picoamperes.

## 3. Results and Discussion

### 3.1. Thermoelectric Measurements Using Hot-Air Stream

After the FIB processing of the micro or nanojunction, the structure was electrically tested in the FIB chamber with the use of Kleindiek probe manipulators. This procedure was performed to check that the structure was electrically active. On the other hand, thermoelectric measurements of the thermovoltage ThV (generated due to a temperature difference between both junctions) were performed outside the FIB system using the Keithley K617 source-meter for the voltage measurements and averaged over five measurements (with an error ±0.05 mV). Firstly, the flow of hot air was used for heating one thermojunction (the “hot” junction; i.e., the left junction). The hot air coming out of the air-heater (equipped with a temperature controller) was a source of heating. This source waschosen due to precise control of the temperature in this case.

A hot airstream (coming out of the nozzle with a 1 mm inner diameter) was directed at the left thermojunction. The distance between the heated structure and the nozzle outlet was about 1 mm. The temperature of the airstream coming from the nozzle was controlled before each measurement using the Pt 100 temperature sensor. The measurements were carried out at hot-air temperatures ranging from 37.5 °C to 100 °C in increments of 12.5 °C (with an error ±0.5 °C). During the heating of the thermojunction with the hot air from the 1 mm wide nozzle outlet, the other thermojunction was located apart at a distance of above 10 mm; i.e., at the ambient temperature (which during the measurements was equal to 22 °C). The other thermojunction (the right junction) was used as a reference junction and was not heated.

**Figure 4:**
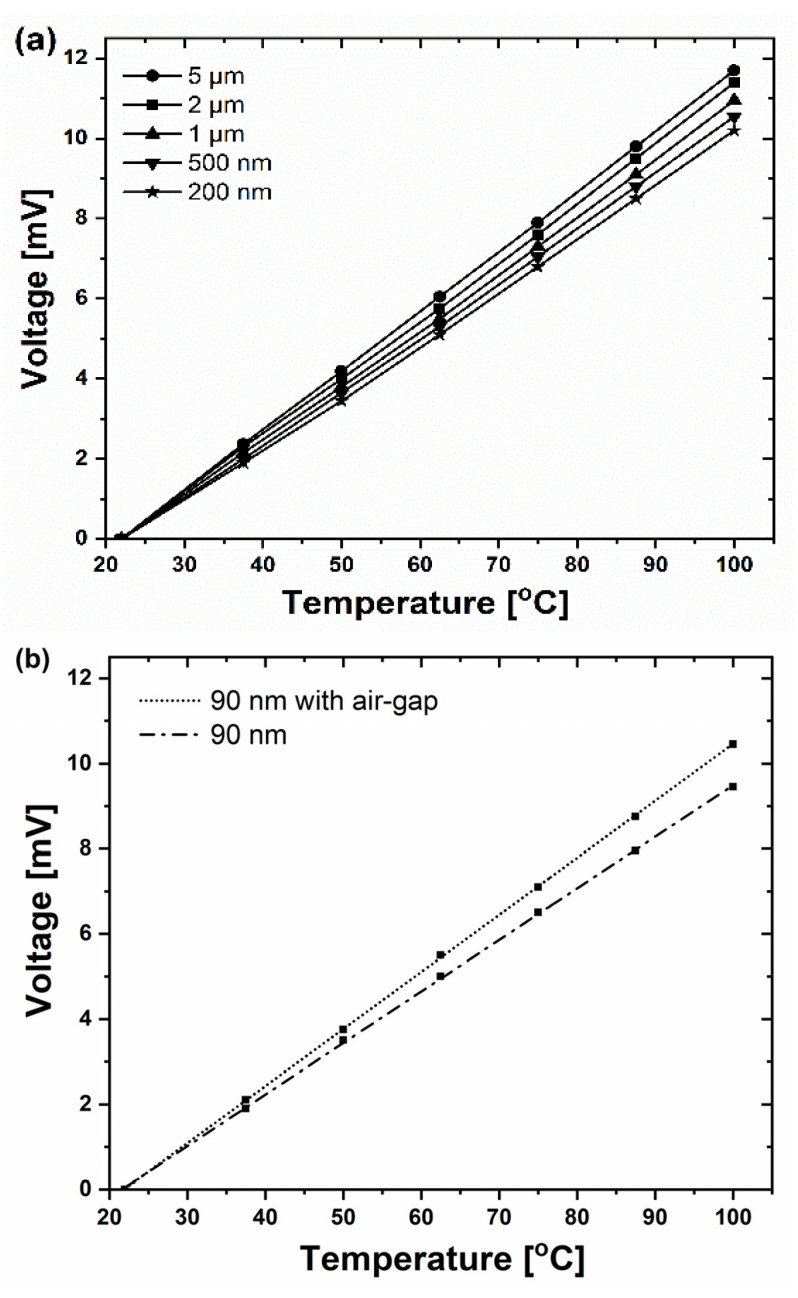
The thermoelectric response of thermocouples fabricated with FIB to heating of the left junction. The results were obtained for (**a**) the left junction (narrowed) with widths ranging from 5 µm to 200 nm, and (**b**) the left junction narrowed to 90 nm width, either without or with the air-gap FIB milled under the nanostructure (i.e., without or with the bridge structure shown in Figure 3f). ThV values were equal to zero at the ambient temperature (22 °C). Measurements of ThV voltages were performed after each narrowing of the left junction.

When the temperature of the airstream increased, a linear rise in recorded voltage (ThV) for all junction widths was observed (Figure 4a). For the prepared micro- or nanostructures, the highest Seebeck coefficients were up to 150 µV/K. This is equivalent to a 10 times larger sensitivity to temperature gradients than obtained from the metal–metal (Pt–W) nanothermocouple described in [24]. The Seebeck coefficients showed a slight increase (Figure 4b) for the case of 90 nm wide nanojunction after milling the air gap under the nanojunction in the way shown in Figure 3f. This effect was due to the reduced volume of the heated material and reduced thermal contact due to removal of a silicon layer.

All the fabricated Pt/Si junctions (micro and nanojunctions) provided an excellent thermoelectric signal detection and linear response, even for the 90 nm wide junction, which enabled nanometer spatial resolution. Moreover, their sensitivity was up to 10 times larger than for typical metal–metal junctions [2,12,24].

### 3.2. Exposure to Laser Beam

**Figure 5:**
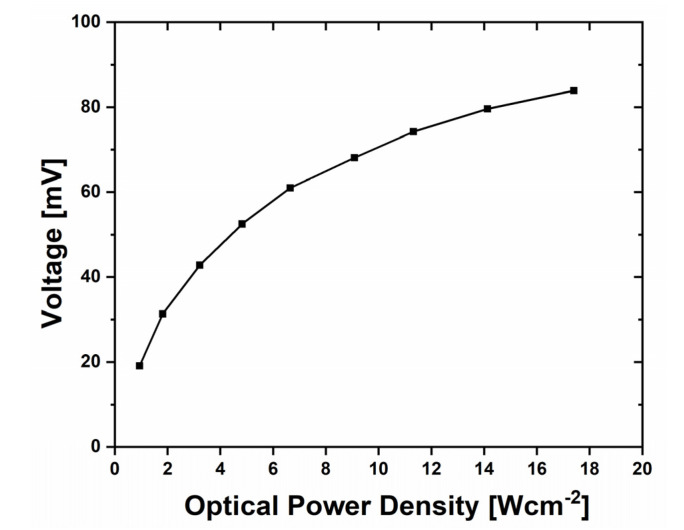
Measurements of the generated voltage recorded during irradiation by an argon-ion laser beam (λ = 514 nm) of the 90 nm structure (with air gap) fabricated by FIB.

To improve the quality of the metal–semiconductor interface of the 90 nm wide nanojunction (in the form of the bridge structure presented in Figure 3f), they were exposed to a powerful beam of an argon-ion laser (with the beam diameter approximately equal to 0.7 mm). The continuous wave (CW) argon laser beam (λ = 514 nm, from H230NDL001 laser unit with variable beam power, produced by National Laser Company) was directed to the thermoelectric nanostructure (the left junction). The optical power density of the laser beam was determined using the ThorLabs PM100D optical power meter with the S130C sensor. The incident laser beam also caused the heating of structure to high temperatures (i.e., the laser annealing of nanojunction), as well as the measured voltage changes at different laser beam powers. The measured voltages for nanojunction were determined using Keithley 2100 multimeter and averaged over 5 measurements (Figure 5).

The experiment with this laser was used to improve the quality of junctions by their annealing and at the same time to determine the response of the structure to the incident laser beam even for the laser beam with an optical power density as high as 17.4 Wcm^−2^, with the signal response equal to 83.85 mV. The observable nonlinearity in Figure 5 can be attributed, among other factors, to a significant heat spreading occurring at high gradients of temperature.

### 3.3. Thermoelectric Measurements Using Hot Airstream after Laser Treatment

After experimenting with the laser illumination, the 90 nm wide nanojunction with an air gap was tested again using a hot airstream. The conditions and parameters of the hot airstream were the same as they were before the laser experiment.

**Figure 6:**
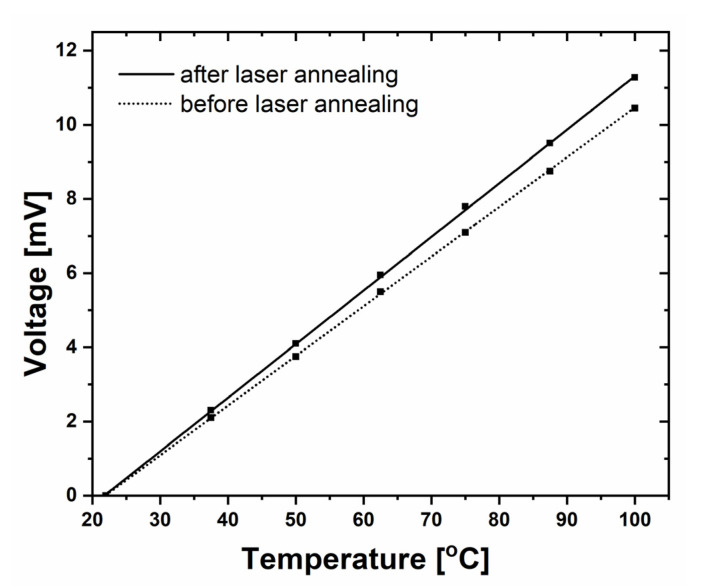
The comparison of thermoelectric (ThV) response of 90 nm nanothermocouple (with air-gap FIB milled under the nanojunction) after experiments using a hot airstream. The measurements were performed before (the dashed line, visible also in Figure 4b) and after (the solid line) the exposure of both left (i.e., later, the hot) and right (i.e., later, the cold) junctions to the laser beam (and thus their annealing).

The obtained ThV results improved when both junctions (left and right) had been previously exposed to the powerful laser beam; i.e., when laser annealed (Figure 6). The improvement was related to higher Seebeck coefficients (and therefore also ThV values). The Seebeck coefficient for the laser-heated nanojunction increased to 140 µV/K, while it was equal to 135 µV/K before the laser heating. Generally, it is important to apply high-temperature annealing with the aim of increasing the quality of metal–semiconductor electrical contact after the FIB deposition of a metal layer on a semiconductor. As shown, the application of laser annealing as described above was an easy and effective method to fulfil this need.

For different cases of FIB deposition (various metals, semiconductors, and details of the deposition process), the improvement can be even larger due to annealing. This improvement was most likely related to the quality improvement of metal-semiconductor interface in nanojunctions during the laser heating. The annealing does not influence the linearity of obtained results (as shown in Figure 6), and the measurements give reproducible results.

The voltage response of each nanojunction may be measured when applying preprogrammed temperatures (e.g., with a hot airstream). Thus, such calibrated nanothermocouples can also be effectively used in the case of a multijunction set, enabling precise measurements even if small differences between their individual responses (i.e., their Seebeck coefficients) occur. Then, the “cold” (unheated) junction is common for all nanojunctions.

### 3.4. Application of Thermoelectric Nanothermocouples Fabricated by FIB

The use of the thermoelectric nanostructure presented in this work to study the laser beam may be particularly useful for a highly sensitive analysis of the optical near-field distribution and of the mode structure of laser beams with nanometer resolution and over a wide spectrum of wavelengths. The point-to-point scanning of the whole area of the laser beam in the near-field using thermoelectric nanostructures can determine the nature of the laser beam; in particular, the spatial intensity distribution of the emitted radiation. Such an approach would allow for the experimental study of the phenomenon of the fibrous structure formation within the laser beam or enable the control of the beam-steering phenomenon [29,30].

An important advantage of such research is the possibility of designing structures with intentionally increased losses for unwanted modes in a laser beam. Such an example for a mid-infrared semiconductor laser has been presented [31] where the higher order modes were removed. The available measurements, however, were performed and shown only for the far-field pattern of the device (and with 1 mm intervals between the measurement points).

Other potential applications of thermoelectric nanostructures in near-field research are, e.g., optimizations of the threshold current and the wall-plug efficiency and improvements of the optical power or the luminance quality for almost all types of lasers.

## 4. Conclusions

The research aimed at manufacturing a metal–semiconductor (Pt–Si) nanothermocouple by the FIB technique and determining the level of its thermoelectric response. The obtained results confirmed that the nanothermocouple can be suitable for the study of laser beams with a nanometer spatial resolution and high ssensitivity (10 times better than for metal–metal nanostructures). A Seebeck coefficient up to 140 µV/K was measured, with a highly linear response to heating temperatures. An experiment with the junctions exposed to a powerful argon-laser beam was performed to improve the quality of junctions (by laser annealing) and to determine the response of the structure to such a high-power incident laser beam. The measurements performed after the abovementioned process revealed an improvement of the nanothermocouple parameters, related to the quality increase of the metal–semiconductor interfaces in the junctions. Our nanothermocouple based on a metal–silicon nanojunction (Pt–Si) was intended for the study of low-energy laser radiation, which causes small changes in temperature; therefore, the operating temperature range of our thermocouple should be in the range from ambient temperatures up to 100 °C.

The obtained results show the potential usefulness of nanothermocouples made with the FIB technique. They can be used particularly in specialized thermoelectric sensors/detectors with nanometer spatial resolution for detecting the heat and radiation of various spectral ranges and intensities, especially for the near to far infrared range.

In contrast to the fabrication of thermometric structures, which requires a high-tech semiconductor manufacturing line with sophisticated fabrication techniques, environments, and many pieces of advanced equipment, our work shows the results of the fabrication of nanothermocouples (with excellent parameters) using the FIB method, which is available in many research laboratories (i.e., without cleanrooms and other high-tech environments) and can be performed by a single operator. Improvements of the FIB technique should lead to even higher Seebeck coefficient of the nanothermocouples fabricated with this method; e.g., in case of the availability of other suitable metal sources (e.g., of chromium, as applied in [18]).

## Data Availability

Not applicable.
